# Efficacy of Natural β-Carotene Chewable Tablets Derived from Banana (*Musa* AA) Pulp in Reducing UV-Induced Skin Erythema

**DOI:** 10.3390/nu17010065

**Published:** 2024-12-27

**Authors:** Chatnarong Putthong, Thanasorn Panmanee, Pensri Charoensit, Sukunya Ross, Kongaphisith Tongpoolsomjit, Jarupa Viyoch

**Affiliations:** 1 Department of Pharmaceutical Technology, Faculty of Pharmaceutical Sciences and Center of Excellence for Innovation in Chemistry, Naresuan University, Phitsanulok 65000, Thailand; chatnarongp@nu.ac.th (C.P.); thanasornp66@nu.ac.th (T.P.); pensric@nu.ac.th (P.C.); 2 Department of Chemistry, Center of Excellence in Biomaterials, Faculty of Science, Naresuan University, Phitsanulok 65000, Thailand; sukunyaj@nu.ac.th; 3 Department of Industrial Chemistry, Faculty of Applied Science, King Mongkut’s University of Technology North Bangkok, Bangkok 10800, Thailand; kongaphisith.t@sci.kmutnb.ac.th

**Keywords:** nutraceutical, β-carotene, photoaging, erythema, ultraviolet light

## Abstract

Background/Objectives: UV radiation is a primary cause of skin damage and photoaging. β-carotene, a potent antioxidant, aids in mitigating UV-induced oxidative stress and enhancing skin photoprotection. This research aimed to evaluate the efficacy of a nutraceutical product designed to prevent photoaging. Methods: The product consists of a blend of hemp seed oil and banana (*Musa* AA), formulated as a chewable tablet. Healthy male participants aged 35–50 years were enrolled in a randomized, parallel, single-blind, placebo-controlled clinical trial. Participants received either the chewable nutraceutical (five tablets after meals in the morning and evening, equivalent to 8 ± 2 mg/day of β-carotene and 400 mg/day of PUFA) or a chewable placebo for 16 weeks. A total of thirty-six participants successfully completed the entire 16-week study. Results: Administration of the nutraceutical resulted in a significant reduction (*p* < 0.05) in UV solar light stimulator-induced erythema on the dorsal skin at week 4, with a mean value of 3.76 ± 0.46 AU, compared to the initial value of 4.88 ± 0.62 AU at week 0. Additionally, serum β-carotene concentration significantly increased from 0.45 ± 0.02 µg/mL at week 0 to 0.61 ± 0.06 µg/mL at week 16 (*p* < 0.05). Moreover, skin intensity in the sun-exposed arm area also significantly improved at week 16, increasing from 71.33 ± 3.50 at week 0 to 81.80 ± 4.45 (*p* < 0.05). Conclusions: The results indicate that the developed nutraceutical may offer effective protection against erythema, making it a promising option for preventing photoaging.

## 1. Introduction

Skin health, particularly protection against UV-induced damage, has gained increasing attention due to growing concerns about skin cancer, premature aging, and other photo-induced skin conditions. Ultraviolet (UV) radiation, specifically UV-B (280–320 nm), is a primary factor in inducing erythema, an inflammatory skin response characterized by redness and swelling. This response occurs when UV-B exposure damages cellular DNA, triggering the release of inflammatory mediators, including prostaglandins, cytokines, and histamines. These compounds increase local blood flow, resulting in visible erythema [1,2]. Dietary interventions, particularly those rich in antioxidants capable of neutralizing UV-generated free radicals, represent a promising approach to mitigating UV damage. Among these antioxidants, β-carotene has been extensively studied for its photoprotective properties and its role in reducing UV-induced erythema and other forms of skin damage [3,4].

In Thailand, several banana cultivars serve as important crops for both local and export markets, particularly the *Musa* AA group, notably “Kluai Khai”. Previous research has demonstrated that Kluai Khai fruits contain significant concentrations of β-carotene, ranging from 3000 to 5000 µg/100 g of fresh fruit, varying with harvest time and ripeness stage [5]. Studies have shown that administering fresh ripe banana fruit to mice (1 mg/g body weight/day, orally for 12 weeks) protected against dermal connective tissue degradation and helped maintain skin glutathione levels following UVB radiation exposure [6,7]. Current evidence suggests that long-term β-carotene supplementation, at doses up to 15 mg daily, can provide photoprotective benefits without adverse effects [8].

As a fat-soluble phytonutrient, β-carotene exhibits low bioavailability [9]. A critical factor affecting its absorption is bioaccessibility, defined as the fraction of carotenoids potentially available for intestinal absorption through micelle formation [10]. Therefore, developing formulations that enhance β-carotene release from the food matrix, such as chewable forms, may improve its bioaccessibility. Additionally, incorporating oils in the formulation can enhance intestinal absorption by facilitating the formation of β-carotene-containing oil droplet micelles, a crucial step in β-carotene absorption.

Hemp seed oil is abundant in linoleic acid (LA, 18:2 *n*-6) and α-linolenic acid (ALA, 18:3 *n*-3), constituting its principal sources of omega-6 and omega-3 polyunsaturated fatty acids (PUFAs), respectively, which might facilitate micelle formation in the gut. The ideal ratio of omega-6 to omega-3 fatty acids in hemp seed oil, often 3 to 4:1, is significant from a nutritional perspective. Consequently, the inclusion of alternative oils like hemp seed oil, which contains these advantageous PUFAs, in conjunction with a main supply of β-carotene from natural sources, is likely to promote micelle formation, thereby improving β-carotene absorption. Moreover, hemp seed oil has natural antioxidants such as tocopherols, which protect β-carotene from oxidative destruction [11,12].

According to the above information, we hypothesized that consuming natural sources of β-carotene from banana pulp, which was formulated into chewable tablets, plus hemp seed oil to facilitate β-carotene absorption may serve as a convenient way to enhance skin protection against UV radiation. To improve this hypothesis, we investigated the efficacy of chewable tablets made from banana pulp containing natural β-carotene in reducing UV-induced skin erythema. The hemp seed oil was added into a formula to facilitate β-carotene absorption. The serum β-carotene was also determined to observe the relationship between the β-carotene level in serum and the photoprotective effects. The obtained data can determine if the natural β-carotene from banana (*Musa* AA) can serve as a photoprotective nutrient against UV-induced skin erythema.

## 2. Materials and Methods

### 2.1. Product Preparation

In accordance with our previous study [13], the chewable tablets were formulated using banana powder made from Musa AA group (Kluai Khai) bananas, sourced from natural banana farms in Lower Northern Thailand. The banana powder was produced by freeze-drying ripe banana pulp, retaining its bioactive compounds, including β-carotene, which was analyzed using RP-HPLC. The analysis revealed that the powder contained approximately 900–1200 µg of β-carotene per gram [13]. Hemp seed oil, cold-pressed from dehulled seeds, was included as a source of polyunsaturated fatty acids (PUFAs), with an omega-6 to omega-3 ratio of approximately 4:1 [8,13]. The tested chewable tablet (TCT), weighing 1200 mg, consisted of 70% *w*/*w* dried banana fruit powder as the major ingredient and 5% *w*/*w* hemp seed oil. Other excipients, including absorbents, diluents, binders, and lubricants, were added to facilitate tableting [5,13,14]. Each tablet provided 0.8–1.0 mg of β-carotene and approximately 40 mg of PUFAs. The placebo chewable tablet (PCT) had a similar composition, except fillers and other pharmaceutical excipients were used instead of banana fruit powder and hemp seed oil.

### 2.2. Efficacy Evaluation

#### 2.2.1. Clinical Study Design

The research study adhered to the ethical principles outlined in the Declaration of Helsinki. The study protocol received approval from the Naresuan University Institutional Review Board (NUIRB) (COA No. 322/2021; Period: 29 July 2021 to 29 July 2022; First Consecutive Certification Date: 29 July 2022 to 29 July 2023). The study was a randomized, parallel, single-blind, and placebo-controlled design and was conducted at the Cosmetics and Natural Products Research Center (CosNat), Naresuan University, from February to September 2022. Prior to participation, all individuals received written information about the study and provided signed informed consent. Only those participants who met the inclusion and exclusion criteria were enrolled. The enrolled participants were then randomly allocated to receive either chewable tablets containing dried banana fruit pulp and hemp seed oil (referred to as the tested chewable tablet, TCT) or placebo chewable tablets (PCTs), with random codes assigned using a predetermined table (e.g., ASXA for treatment for person 1, ZWER for placebo for person 1, TTIO for treatment for person 2, VCXF for placebo for person 2, and so on). An independent staff member distributed the products labelled with codes to enrolled participants. The randomization codes remained concealed from all investigators until the completion of data analysis. Skin erythema levels were measured using a chromameter on UV-exposed skin on the back, and skin structure was assessed on the forearm using ultrasound before (week 0) and after product administration for 16 weeks. Additionally, serum β-carotene content was measured before and after product administration.

#### 2.2.2. Participants

In this study, the participants were recruited through advertising. Forty healthy Thai male participants meeting specific inclusion and exclusion criteria were enrolled. The inclusion criteria were as follows: participants had to be healthy males aged between 35 and 50 years, with a body mass index (BMI) ranging from 18 to 29 kg/m^2^. Additionally, participants were required to have a Fitzpatrick skin classification of type III or IV (indicative of Asian skin color) [15], with uniformly colored back areas and arms. For exclusion criteria, candidates were ineligible if they had wounds or scars in the evaluation area, a history of allergic reactions to ingredients found in products, including bananas or hemp seed oil, or to any other excipients used for tabletting purposes. Additionally, individuals with a history of dermatitis, eczema, or psoriasis within the past 6 months or a history of skin cancer within the last year were excluded. Those who had used steroids, antibiotics, nonsteroidal anti-inflammatory drugs, or antihistamines within 3 days prior to the study or topical medicines in the evaluation area were also excluded. Furthermore, participants with a history of major surgery within the past year, regular sunbathing at least once a week, or current tobacco or alcohol use (smoking at least one cigarette daily or consuming at least one glass/250 mL of alcohol daily) were excluded. During the study, participants were prohibited from using skincare products with properties such as skin polishing, brightness enhancement, black spot reduction, or moisturization for the back and arms.

#### 2.2.3. Study Procedures

Before beginning the study, the erythema response to UV light of each participant was determined as the minimal erythema dose (MED). On the first day of the study, the enrolled participants had their weight, height, and vital signs (including blood pressure (mm/Hg), heart rate (beats/min), and body temperature (°C)) measured to determine their BMI and health status. The skin structure of their right forearm (a sun-exposed side) was then measured. After that, they were irradiated with UV light at 1.5 MED on a selected area of their back. Their skin color was measured before and 24 h after UV exposure to evaluate UV resistance. Additionally, blood samples were corrected.

The total 40 enrolled participants were divided into 2 groups, with 20 participants in each: the TCT group (participants receiving the tested product, TCT) and the PCT group (participants receiving the placebo product, PCT). They were given the coded products in amounts sufficient for each visit duration (visit at 4, 8, and 16 weeks after administration), along with administrative instructions. The participants were instructed to administer the product 5 tablets at a time, in the morning and evening after meals, with a total intake of 8 ± 2 mg/day of β-carotene and not more than 1000 mg/day of PUFAs per participant. During the 16 weeks of the study, participants were not required to modify their dietary habits. However, they were expected to routinely document the specific types and quantities of food consumed at each meal. Additionally, the participants were given a book to record the daily administration time of the products and any undesirable symptoms. The details of the measurement of each parameters, including UV resistance, skin structure, blood components, and β-carotene content in serum, are as follows:

##### UV Resistance Assessment

The minimal erythema dose (MED) of UV radiation for each participant was determined by irradiating a 2 × 2 cm area on their back using a UV Solar Light Stimulator (Model 601 V 2.5, Solar Light Company, Glenside, PA, USA). To measure the erythema response of the irradiated skin, the selected area was irradiated with 1.5 times the MED. Skin color was assessed before and 24 h after exposure using a Chroma Meter (Minolta CM-700d, Osaka, Japan). The obtained a+ (red color) values before (a0) and after exposure (a24) were recorded. A lower difference between a24 and a0 (a24-0) indicates lower skin redness (erythema) and higher resistance to UV light. The resistant assessment was performed at 0, 4, 8, and 16 weeks of product administration.

##### Skin Structural Assessment

Skin structure analysis of the right forearm exposed to sunlight was conducted using a non-invasive method, specifically, ultrasound (Dermalab Combo, Cortex Technology, Aalborg, Denmark). Skin structural intensity was determined using a pulsed 20 MHz B-mode high-frequency ultrasound, with a penetration depth of 3.4 mm, which allowed differentiation of the echogenic dermis from the hypoechoic subcutaneous tissue [16], presented as an arbitrary unit. In this study, skin intensity was measured at baseline (0 weeks) and at 4, 8, and 16 weeks after administration.

##### Blood Sample Collection and Analysis

Blood samples were obtained at weeks 0 and 16 to assess blood components, including creatinine, LDL, HDL, fasting glucose, potassium, and sodium. The serum derived from the blood sample was stored at a temperature of −80 °C until it underwent analysis using reversed phase high-performance liquid chromatography (RP-HPLC) methods to determine the concentration of β-carotene. For β-carotene analysis, 100 µL of serum was transferred to a test tube, and 100 µL of absolute ethanol was added. The contents were agitated vigorously for a few seconds, 200 µL of hexane was added, and the mixture was agitated again using vortex mixing for 30 s. The mixture was centrifuged for 1 min at 3000 rpm. Subsequently, a pipette was used to transfer 150 µL of the top layer into a test tube. The hexane was evaporated under a speed vacuum. The residue was redissolved in 100 µL of absolute ethanol and filtered through disposable 0.45 µm filters prior to analysis via HPLC (Shimadzu LC-20AT system): c18 column; 10% EtOAc:MeOH (1:1) in acetonitrile; flow rate: 2 mL/min; UV-visible detector wavelength: 450 nm. β-Carotene in all samples was quantified using a freshly prepared standard β-carotene (≥93% purity, Standard grade, Sigma-Aldrich, St. Louis, MO, USA) as a standard curve. The HPLC system was controlled, and data were collected and integrated using LC solution software (version 1.24 SP1, Shimadzu, Japan). The modified HPLC method was validated according to the previous report using the determination of analytical parameters: linearity range, limit of detection (LOD), limit of quantification (LOQ), precision, and accuracy [17].

##### Compliance

Participants were asked to keep a diary to record product usage and any symptoms experienced during the study period. Investigators reviewed these diaries to assess compliance and document adverse events. At each visit, participants were verbally queried about the frequency and timing of product administration. Compliance was also monitored by checking the amount of product remaining.

#### 2.2.4. Statistical Analysis

Statistical analysis included the determination of mean values, standard deviation (SD), and standard error of the mean (SEM), as well as the use of Student’s *t*-tests to compare two independent groups. Additionally, variability analysis, specifically analysis of variance (ANOVA), was used for multiple comparisons. The statistical significance level was set at a 95% confidence level, denoted by a *p*-value threshold of less than 0.05.

## 3. Results and Discussion

### 3.1. Baseline Characteristics of the Participants

Forty healthy male participants were recruited. Among the participants, 20 individuals were randomly assigned to receive TCT, while the remaining 20 were assigned to receive PCT. After three to four weeks, four participants withdrew from the study. Notably, all four individuals belonged to the PCT group; withdrawal was possibly due to a dislike of the PCT’s taste. The health status data, including age, BMI, blood pressure, heart rate, and body temperature, along with laboratory blood test results, from the withdrawn participants were not included in the study. Table 1 shows the health status data and mean baseline characteristics of the 36 remaining participants (20 in the TCT group and 16 in the PCT group). The enrolled participants ranged in age from 35 to 49 years, with an average age of 41.75 ± 4.09. Their BMI averaged 24.07 ± 2.81 Kg/m^2^. There were no significant differences in health status and blood components between the two groups (*p* > 0.05).

### 3.2. Effectiveness Against UV-Induced Erythema

#### 3.2.1. UV Resistance

In this study, we evaluated the efficacy of TCT in providing UV resistance. We measured the a24-0 h value, which represents the change in skin redness before and 24 h after UV exposure on the skin of participants’ backs. Participants received either TCT (with a total intake of 8 ± 2 mg/day of natural β-carotene and not more than 1000 mg/day of PUFAs) or PCT (placebo) products over 0, 4, 8, and 16 weeks. Lower a24-0 h levels indicate reduced inflammation and skin erythema, reflecting higher resistance to UV radiation in the exposed skin area. This increased resistance helps prevent photoaging, as skin inflammation significantly contributes to skin aging [4].

**Figure 1:**
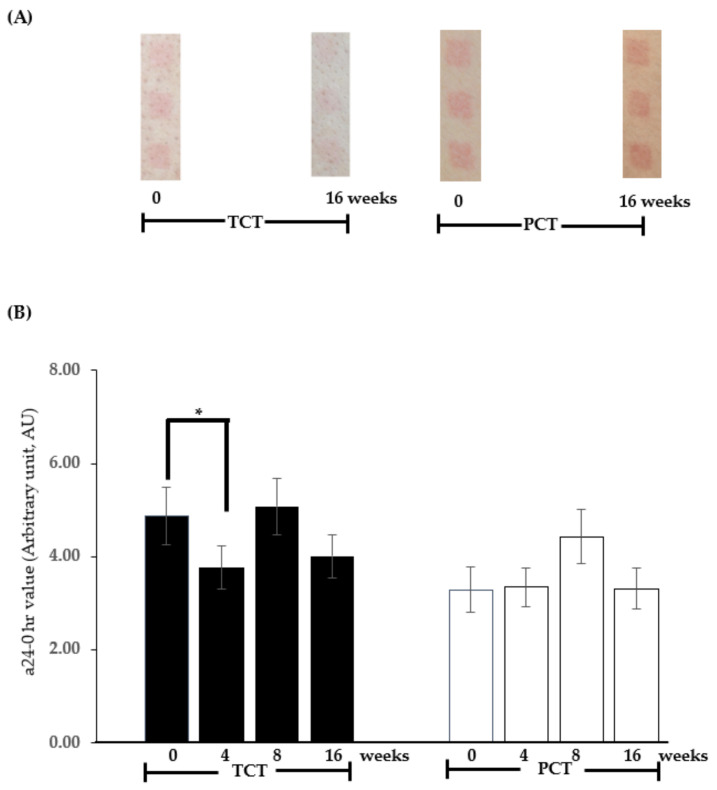
Sample photos of the redness level on the UV-exposed skin areas of the participants’ backs 24 h after radiation at 0 and 16 weeks post-administration of the tested chewable tablet (TCT) and placebo chewable tablet (PCT) (**A**). The mean ± SEM of the a24-0 h values (arbitrary units, AU) on the UV-irradiated skin area of participants receiving TCT (dark bar) or PCT (light bar) over 0, 4, 8, and 16 weeks is shown in (**B**). * *p* < 0.05, paired two-tailed *t*-test, compared to the 0-week baseline within each respective group.

Figure 1A shows sample photos of the redness level on the UV-exposed skin areas of the participants’ backs 24 h after radiation, at both baseline (0 weeks) and 16 weeks post-TCT and PCT administration. As shown in Figure 1B, a statistically significant (*p* < 0.05) decrease in a24-0 h value was observed in the TCT group after 4 weeks of administration (4 weeks, mean ± SEM: 3.76 ± 0.46 AU) compared to baseline (0 weeks, 4.88 ± 0.62 AU). In contrast, no significant decrease was observed in the PCT group at any time point. At 8 weeks, both groups exhibited an increase in a24-0 h values, reflecting a temporary spike in UV-induced inflammation. This unexpected variation is attributed to uncontrolled external factors, such as seasonal activities and increased UV exposure in April (summer in Thailand). By 16 weeks, the a24-0 h values decreased again in both groups, particularly in the TCT group, where the value was lower than at baseline (16 weeks, 4.01 ± 0.46 AU). However, this difference was not statistically significant compared to baseline (*p* = 0.0823). These findings suggest that a diet rich in β-carotene (8 ± 2 mg/day), along with omega-6 and omega-3 fatty acids at a daily intake of approximately 400 mg in a 4:1 ratio, potentially enhances UV resistance, thus mitigating skin aging processes from UV exposure [18].

The photoprotective effects of β-carotene are primarily attributed to its antioxidant capacity, quenching free radicals generated in the skin after UV exposure [19]. Our previous study using a UVB-irradiated mouse model demonstrated that consumption of banana fruit enriched with natural β-carotene could enhance the production of endogenous antioxidants, such as glutathione, by stimulating the expression of γ-glutamylcysteine synthetase, a rate-limiting enzyme for glutathione synthesis [20].

Clinically, photoprotective effects of β-carotene have been reported in previous studies [21]. However, inconsistencies exist among studies, depending on formulation, dose, and duration of administration. For instance, no photoprotective effect was observed in participants given an oral administration of a 120 mg single dose or 90 mg/day of β-carotene for 3 weeks [21]. In contrast, decreased erythema induced by natural sunlight was found in participants supplemented with 30 mg β-carotene for 10 weeks, and the combination of oral supplement and sunscreen administration synergized the photoprotective effects [22]. These results suggest that administration of a low dose for a longer period, such as at least 10 weeks, may provide the beneficial effects of β-carotene with regard to sunlight protection [21].

The recommended dietary intake of β-carotene for adults is approximately 10 mg/day, based on the Recommended Dietary Allowances (RDAs) for vitamin A. The RDA for vitamin A is 900 µg RAE (Retinol Activity Equivalent), with 1 µg RAE equivalent to 12 µg of dietary β-carotene [23]. Interestingly, our study showed that the photoprotective effect, indicated by decreased erythema levels, began to appear after just 4 weeks of administering a total intake of 8 ± 2 mg/day of dietary β-carotene from banana fruit pulp. This earlier onset of effects compared to previous reports may be due to differences in the source of β-carotene and formulation, which influence β-carotene bioavailability.

In our study, natural β-carotene was sourced from dried banana fruit pulp enriched with β-carotene, as previously described [4]. The incorporation of hemp seed oil into the formulation likely facilitated the formation of β-carotene-containing oil droplets during mastication and digestion. This oil droplet formation is a critical initial step in the absorption of fat-soluble nutrients, including β-carotene, preceding the formation of oil-in-water micelles necessary for carotenoid transport across enterocytes. Subsequently, β-carotene and its metabolites, such as retinol and retinyl esters, are transported to the skin. At the cutaneous level, β-carotene undergoes conversion to retinal, which can be further oxidized to retinoic acid or reduced to retinol. These compounds exhibit antioxidant activity, potentially counteracting free radicals generated immediately following UV exposure and/or inducing the production of endogenous antioxidants [24]. Consequently, the presence of these compounds might attenuate the downstream cascade involving inflammatory responses and erythema formation.

Moreover, banana fruit pulp contains phenolics [4], while hemp seed oils provide not only polyunsaturated fatty acids (PUFAs), including omega-6 and omega-3 in ratios beneficial for health effects such as anti-inflammation [25], but also various forms of vitamin E [26]. Recent research has shown that oral administration of a combination of carotenoids and vitamin E can more effectively suppress erythema formation compared to carotenoids alone [27]. Therefore, the presence of phenolics from banana fruit pulp and vitamin E from hemp seed oil may have additive or synergistic antioxidant effects with β-carotene. This combination could explain the observed photoprotective efficacy after 4 weeks of administration of the TCT.

#### 3.2.2. Skin Structure

Decreased skin density is a hallmark of photoaging. In UV-exposed areas, overexpression of matrix metalloproteinases (MMPs), particularly MMP-1, has been well-documented [28]. This overexpression results from the activation of signaling pathways in dermal fibroblasts, triggered by generated free radicals and/or inflammatory cytokines released from epidermal keratinocytes or fibroblasts themselves [29]. Based on previous reports demonstrating the ability of β-carotene metabolites to suppress UV-induced MMP overproduction in skin [19], we hypothesized that improved skin density would be observed in this study.

**Figure 2:**
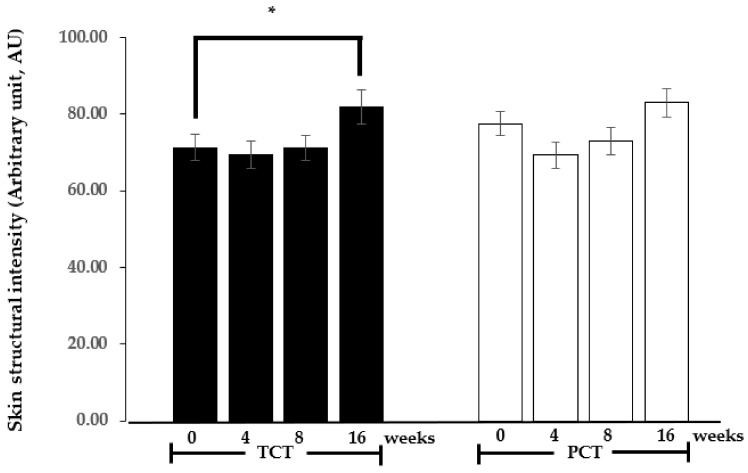
Mean ± SEM of the skin structural intensity (arbitrary units, AU) on the right forearm (sun-exposed area) of participants in groups receiving the tested chewable tablet (TCT, dark bar) or placebo chewable tablet (PCT, light bar) over 0, 4, 8, and 16 weeks. * *p* < 0.05, paired two-tailed *t*-test, compared to the 0-week baseline within each respective group.

Figure 2 illustrates the results of the structural skin analysis, which measured skin density in the sun-exposed area of the forearms. No statistically significant difference (*p* > 0.05) was observed in the skin density of participants who received the PCT for 16 weeks (16 weeks, mean ± SEM: 82.96 ± 3.76) compared to the baseline values (0 weeks, 77.56 ± 3.12). However, statistical analysis revealed a significant increase (*p* < 0.05) in skin density values within the TCT group (16 weeks, 81.80 ± 4.45) compared to the initial values (0 weeks, 71.33 ± 3.50). The changes observed in the TCT group suggest that daily consumption of the product containing 8 ± 2 mg of β-carotene, along with 400 mg of omega-6 and omega-3 fatty acids at a ratio of 4:1, influences the structural constituents of the skin. These results align with a prior investigation involving 30 participants who received a daily intake of a higher amount of β-carotene, 30 mg/day over 90 days, which resulted in significant improvements in skin flexibility and strength and a reduction in wrinkles [21]. The improvement in skin properties was attributed to increased expression of type I collagen mRNA.

Notably, the photoprotective activity of TCT in minimizing erythema after UV exposure was observed at 4 weeks post-administration, while improvement in skin density in the sun-exposed area was evident at 16 weeks. This difference in efficacy timeline may be attributed to distinct mechanisms of action. The immediate antioxidant activities, which minimize acute inflammation and erythema after UV exposure, may be sufficient after 1 month of TCT administration. During this period, β-carotene and its active metabolites can protect against free radical-induced damage to skin macromolecules, including DNA, lipids, and proteins, particularly in the connective tissue of the dermal layer. However, improving skin density requires not only prevention of free radical-induced damage but also stimulation of downstream mechanisms resulting from active metabolites of β-carotene accumulated in the skin. These mechanisms include the effects of retinoic acid on suppressing collagenases and inducing type I procollagen synthesis [30]. The increased accumulation of collagen leading to improved skin density requires a specific duration of treatment. This temporal difference explains the observed timeline discrepancy between the onset of photoprotective effects and improvements in skin density.

### 3.3. Level of Serum β-Carotene and Other Blood Components

**Figure 3:**
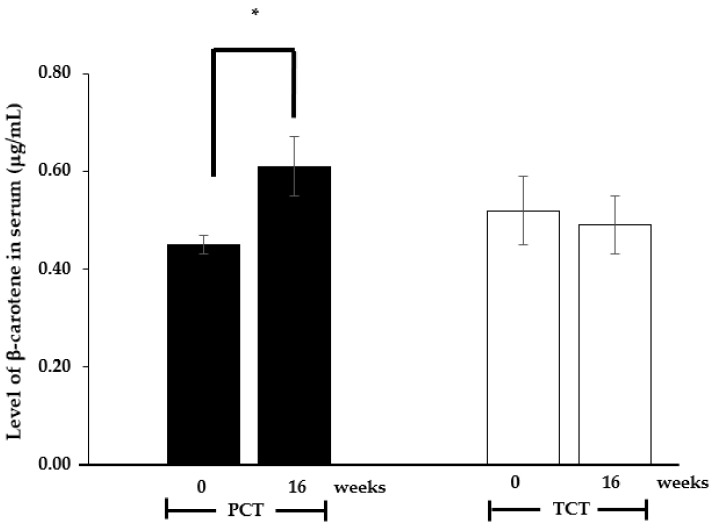
Mean ± SEM of level of β-carotene (μg/mL) in serum of participants in groups receiving the tested chewable tablet (TCT, dark bar) or placebo chewable tablet (PCT, light bar) over 0 and 16 weeks. * *p* < 0.05, paired two-tailed *t*-test, compared to the 0-week baseline within each respective group.

Serum β-carotene levels in participants were quantified before and after 16 weeks of product administration. The results are presented in Figure 3. This study demonstrated that regular consumption of the TCT resulted in a statistically significant increase in serum β-carotene concentrations (*p* < 0.05). Specifically, an estimated 26% increase in serum β-carotene concentration was observed, with values rising from 0.45 ± 0.02 μg/mL at baseline to 0.61 ± 0.06 μg/mL at the end of the 16-week intervention period. No significant difference (*p* > 0.05) was detected in serum β-carotene levels between the group of participants who received the PCT for 16 weeks (0.49 ± 0.06 μg/mL) and the baseline measurement (0 weeks, 0.52 ± 0.07 μg/mL). These findings are consistent with previous research reporting elevated serum β-carotene levels following a 12-week administration of a 25 mg/day carotenoid supplement.

The observed elevation in β-carotene concentrations within the serum correlates with an increase in skin density. As previously mentioned, the formulation composition of TCT facilitates β-carotene absorption through the intestine. The trend towards higher serum β-carotene concentrations in the TCT group compared to the PCT group suggests that the formulated TCT effectively releases β-carotene and promotes its absorption into the bloodstream. It is well established that following digestion of carotenoid-enriched foods, carotenoids such as β-carotene must be released from the food matrix and form micelles in the gastrointestinal tract for transport to enterocytes, as discussed earlier. The chewable tablet formulation likely enhances β-carotene release through a combination of mechanical force and enzymatic reactions. Upon absorption into enterocytes, β-carotene is either incorporated into chylomicrons for exocytosis or enzymatically cleaved to retinol before undergoing esterification to retinyl esters and subsequent packaging into chylomicrons. In humans, approximately 60% of ingested β-carotene is converted to retinol [23]. Chylomicrons containing β-carotene and retinyl esters are released from enterocytes into the bloodstream and directed to the liver. In the liver, β-carotene is either stored or secreted back into the bloodstream, circulating as lipoprotein (primarily low-density lipoprotein, LDL)-associated β-carotene, which is taken up by various tissues, including the skin, where β-carotene produces active metabolites. Consequently, higher levels of serum β-carotene could indicate an increased opportunity for β-carotene uptake by skin tissue, potentially leading to enhanced biological activity of β-carotene metabolites in reducing UV-induced skin erythema and improving skin density.

Blood components, including creatinine, LDL, HDL, fasting glucose, potassium, and sodium were also measured at 16 weeks post-administration of TCT and PCT (Table 2). No significant changes were observed in these parameters when comparing baseline to 16 weeks post-administration in both the TCT and PCT groups. Interestingly, HDL levels in the TCT group showed a tendency to increase. This effect might be attributed to the presence of hemp seed oil in the formula, as previous studies have reported that hempseed oil with an appropriate omega-6 to omega-3 ratio can enhance HDL levels [31].

A limitation of this study is the lack of serum β-carotene measurements prior to the 16-week timepoint. Nevertheless, our findings suggest, at least partially, the effectiveness of the formulated product in reducing UV-induced skin erythema and potentially improving skin structure. Further studies involving a larger cohort of participants, monitoring of serum β-carotene at various timepoints throughout the administration period, and measurement of β-carotene levels in skin tissue should be conducted to elucidate the kinetics of absorption and distribution of natural β-carotene formulated into a chewable dosage form.

## 4. Conclusions

This study demonstrated that chewable tablets containing natural β-carotene, omega-6, and omega-3 fatty acids enhanced UV resistance and skin density in healthy male participants. The results showed significant benefits at week 4, particularly a notable reduction in UV-induced erythema. However, at week 8, both the placebo and test groups experienced a temporary increase in UV-induced erythema, likely due to uncontrolled external factors such as increased seasonal UV exposure. Importantly, in the test group, this increase did not exceed the initial erythema value (measured at week 0), while the placebo group showed higher erythema levels compared to the initial values. This difference suggests that the test product helped resist erythema response. Additionally, after 16 weeks, the treatment group demonstrated increased serum β-carotene levels and improved skin structural intensity, indicating enhanced β-carotene absorption and overall skin health benefits. To fully validate the formula’s efficacy, further long-term studies are needed to confirm consistent effects and better understand β-carotene’s long-term impact on skin health.

## Figures and Tables

**Table 1:** Health status data and laboratory blood test values of the participants who completed the study.

	Mean ± SD		*p* *
	The Tested Chewable Tablet (TCT) (N = 20)	The Placebo Chewable Tablet (PCT) (N = 16)	
Health status			
Age, years Age range	41.70 ± 5.27 (35–49)	41.44 ± 3.76 (35–47)	0.8690
BMI (kg/m^2^)	22.11 ± 5.32	22.66 ± 5.90	0.7708
Blood pressure (mm/HG)			
Systolic	128.4 ± 9.7	125.2 ± 6.7	0.2700
Diastolic	82.5 ± 10.3	78.9 ± 12.3	0.3458
Heart rate (beats/min)	83.9 ± 9.7	86.1 ± 8.3	0.4764
Body temperature (°C)	36.7 ± 0.5	36.8 ± 0.4	0.5200
Laboratory blood test values			
FBS (mg/dL)	104.65 ± 33.91	104.67 ± 22.40	0.9948
LDL (mg/dL)	140.05 ± 27.27	140.36 ± 45.58	0.9799
HDL (mg/dL)	49.60 ± 16.54	41.13 ± 8.79	0.0735
Potassium (mEq/L)	4.06 ± 0.25	3.98 ± 0.23	0.3301
Sodium (mEq/L)	143.75 ± 2.94	143.69 ± 3.20	0.9537

* Unpaired *t*-test (two-tailed *p*-value) for group comparison.

**Table 2:** Laboratory blood test values of the participants before (0 weeks) and after 16 weeks of oral administration of the tested chewable tablet (TCT) or placebo chewable tablet (PCT).

Laboratory Blood Test Values	Mean ± SD			
	The Tested Chewable Tablet (TCT) (N = 20)		The Placebo Chewable Tablet (TCT) (N = 16)	
	0 Weeks	16 Weeks	0 Weeks	16 Weeks
FBS (mg/dL)	104.65 ± 33.91	100.00 ± 33.17	104.67 ± 22.40	99.67 ± 11.47
LDL (mg/dL)	140.05 ± 27.27	135.74 ± 29.06	140.36 ± 45.58	135.07 ± 40.12
HDL (mg/dL)	49.60 ± 16.54	53.05 ± 16.42	41.13 ± 8.79	42.69 ± 6.86
Potassium (mEq/L)	4.06 ± 0.25	3.92 ± 0.19	3.98 ± 0.23	3.78 ± 0.30
Sodium (mEq/L)	143.75 ± 2.94	142.00 ± 1.59	143.69 ± 3.20	140.63 ± 1.75

## Data Availability

The data in this work may be obtained upon request from the first author and corresponding author. The statistics remain inaccessible to the public owing to ethical constraints.
